# Characterization of a novel telomerase-immortalized human endometrial stromal cell line, St-T1b

**DOI:** 10.1186/1477-7827-7-76

**Published:** 2009-07-20

**Authors:** Annemarie Samalecos, Katja Reimann, Stefanie Wittmann, Heinrich M Schulte, Jan J Brosens, Ana-Maria Bamberger, Birgit Gellersen

**Affiliations:** 1 Endokrinologikum Hamburg, 20251 Hamburg, Germany; 2 Institute of Reproductive and Developmental Biology, Imperial College London, Hammersmith Hospital, London W12 ONN, UK; 3 Section on Endocrinology and Ageing, University Clinic Hamburg-Eppendorf, 20251 Hamburg, Germany

## Abstract

**Background:**

Coordinated differentiation of the endometrial compartments in the second half of the menstrual cycle is a prerequisite for the establishment of pregnancy. Endometrial stromal cells (ESC) decidualize under the influence of ovarian progesterone to accommodate implantation of the blastocyst and support establishment of the placenta. Studies into the mechanisms of decidualization are often hampered by the lack of primary ESC. Here we describe a novel immortalized human ESC line.

**Methods:**

Primary ESC were immortalized by the transduction of telomerase. The resultant cell line, termed St-T1b, was characterized for its morphological and biochemical properties by immunocytochemistry, RT-PCR and immunoblotting. Its progestational response was tested using progesterone and medroxyprogesterone acetate with and without 8-Br-cAMP, an established inducer of decidualization in vitro.

**Results:**

St-T1b were positive for the fibroblast markers vimentin and CD90 and negative for the epithelial marker cytokeratin-7. They acquired a decidual phenotype indistinguishable from primary ESC in response to cAMP stimulation. The decidual response was characterized by transcriptional activation of marker genes, such as PRL, IGFBP1, and FOXO1, and enhanced protein levels of the tumor suppressor p53 and the metastasis suppressor KAI1 (CD82). Progestins alone had no effect on St-T1b cells, but medroxyprogesterone acetate greatly enhanced the cAMP-stimulated expression of IGFBP-1 after 3 and 7 days. Progesterone, albeit more weakly, also augmented the cAMP-induced IGFBP-1 production but only after 7 days of treatment. The cell line remained stable in continuous culture for more than 150 passages.

**Conclusion:**

St-T1b express the appropriate phenotypic ESC markers and their decidual response closely mimics that of primary cultures. Decidualization is efficiently induced by cAMP analog and enhanced by medroxyprogesterone acetate, and, to a lesser extent, by natural progesterone. St-T1b cells therefore serve as a useful model for primary ESC.

## Background

Decidualization is a differentiation process of endometrial stromal cells (ESC) that, in humans, is initiated in the secretory phase of the menstrual cycle independently of a blastocyst signal. The transformation of elongated stromal fibroblasts into secretory epithelioid-like decidual cells is first apparent approximately 10 days after the postovulatory surge in circulating progesterone (P4) levels in the vicinity of terminal spiral arteries and then spreads throughout the endometrial compartment through autocrine and paracrine signals. Typical secretory products of decidualized cells are IGFBP-1 and prolactin (PRL). Induction of *IGFBP1 *and decidua-specific *PRL *gene transcription serve as molecular markers of decidualization [1-3]. Furthermore, expression of the forkhead transcription factor FOXO1 is induced with decidualization, both *in vivo *and *in vitro *[4], and levels of the tumor suppressor protein p53 and the tetraspanin KAI1 (CD82), a cell surface protein that functions as a metastasis suppressor in tumor cells, are increased posttranscriptionally in decidualized cells [5-7].

Investigations into the mechanisms of decidualization in humans are largely restricted to cell culture experimentation. Yet such studies are hampered by difficulties in obtaining tissues for the preparation of primary decidual cell cultures. Consequently, attempts have been made to immortalize human endometrial stromal cells by oncogenic transformation. However, SV40-transformed cells display genomic instability [8] and, with time in culture, lose the crucial capability of undergoing decidual differentiation in response to the appropriate stimuli (our own unpublished observations on the St-2 cell line described in [9]). Recently, the use of human telomerase reverse transcriptase (hTERT) as the immortalizing agent for ESCs has yielded more promising results [10,11].

Normal primary cells undergo senescence because linear chromosomes shorten progressively with every round of replication unless the ends of the chromosomes, the telomers, are maintained by telomerase, a reverse transcriptase that adds telomeric repeats de novo after each cell division. While human tumor cells often have aberrantly high telomerase levels, most differentiated adult cells display silencing of telomerase activity, leading to progressive telomere shortening and thus a limited lifespan [12]. The re-introduction of telomerase into primary cells can bestow them with extension of replicative lifespan or even immortalization without the characteristics of neoplastic transformation such as anchorage-independent growth and lack of contact inhibition [13,14]. Telomerase-immortalized ESC lines have been shown to undergo decidualization in response to extended treatment with 17*β*-estradiol (E2) and medroxyprogesterone acetate (MPA), or upon sequential stimulation with epidermal growth factor (EGF), E2 and MPA [10,11].

In the present study, we characterize a new telomerase-immortalized ESC line, termed St-T1b. The ability of these cells to undergo decidualization and their stability in long-term culture are investigated to validate the cell line as a model for primary ESC.

## Methods

### Cell culture

Primary cultures of human ESC were prepared from anonymized uterine biopsy samples obtained from premenopausal women (age 35–50) at the time of hysterectomy for benign gynecological disorders. Purified ESC were prepared as previously described [15] and maintained in ESC medium: phenolred-free Dulbecco's modified Eagle medium (DMEM)/Ham's F12 with 10% steroid-depleted dialyzed fetal bovine serum (FCS) (PAA Laboratories, Cölbe, Germany), 100 U/ml penicillin, 100 μg/ml streptomycin, and 0.2% Primocin (Invivogen, San Diego, CA), and supplemented with insulin (1 μg/ml) and 17*β*-estradiol (E2; 1 nM) (Sigma-Aldrich, Deisenhofen, Germany). Decidualization was performed in minimal medium 1 (MM1; ESC medium without insulin or E2) in the presence of 0.5 mM 8-Br-cAMP (Biolog, Bremen, Germany). The local ethics committee approved this study and patient consent was obtained before tissue collection.

Immortalization of primary ESC was performed in collaboration with Gordon Peters (Cancer Research UK London Research Institute, London, UK). ESC were isolated from a biopsy taken during the proliferative phase from a 30 year old patient with tubal disease awaiting IVF treatment. Briefly, the receptor for ecotropic retroviruses was first stably introduced into the primary ESC using neomycin selection, then the cells were infected with a retrovirus encoding hTERT and selected for hygromycin resistance. The pBABE retrovirus system was used in which the cDNA of interest is driven by the Moloney murine leukemia virus promoter in the viral long terminal repeat and the marker is driven by the SV40 promoter [16]. St-T1b cells were maintained and decidualized in the same media as described for ESC, but in the absence of Primocin. Stimulations with P4, MPA, dexamethasone (all from Sigma-Aldrich) and R5020 (Promegestone; PerkinElmer, Rodgau, Germany) were performed in MM1. With the exception of the long-term observation, all experiments were performed repeatedly between passage numbers 30 and 53, and representative experiments are shown.

The trophoblastic hybridoma cell line AC-1M88, a fusion of primary extravillous trophoblast from term placenta with a mutant of the JEG-3 choriocarcinoma cell line, was kindly provided by H.G. Frank and P. Kaufmann (University Hospital Aachen, Germany) [17,18], and was maintained in DMEM/Ham's F12 with 10% complete FCS, 100 U/ml penicillin, and 100 μg/ml streptomycin.

### Immunocytochemistry

Cells grown on 8-well chamber slides (BD Biosciences, Heidelberg, Germany) were fixed with 4% paraformaldehyde, blocked for endogenous peroxidase activity (0.75% H_2_O_2 _in methanol for 10 min at room temperature), and then immunostained with mouse monoclonal antibodies to vimentin (Clone V9, 1:1,000) (Dako, Hamburg, Germany), CD90 (Clone AS02, 1:100) (Dianova, Hamburg, Germany), or cytokeratin-7 (CK7) (Clone OV-TL, 1:200) (Dako). Immunoreactivity was visualized with 3,3'-diaminobenzidine tetrahydrochloride (DAB; brown) (Vectastain ABC-Elite-Universal:HRP Kit; Linaris).

### Actin staining with FITC phalloidin

Cultured cells were fixed with 4% paraformaldehyde for 10 min at room temperature, washed with PBS for 10 min, and permeabilized with 0.1% Triton X-100 in PBS for 10 min. Following three washes in PBS for 10 min each, cells were incubated in PBS/1% BSA for 25 min. Fluorescein-phalloidin (Invitrogen, Karlsruhe, Germany) stock solution (200 U/ml in methanol) was diluted 1:40 in PBS/1% BSA and placed on the monolayer for 20 min. After three washes in PBS, cells were mounted in ProLong Gold antifade reagent (Invitrogen).

### RNA extraction and RT-PCR

RNA was extracted from cultured cells with peqGold RNApure reagent (Peqlab, Erlangen, Germany) according to the manufacturer's protocol, but the aqueous phase obtained after chloroform extraction was subjected to an additional purification step by phenol/chloroform/isoamylalcohol extraction. One μg of RNA was used for oligo(dT)-primed cDNA synthesis with the ImProm-II Reverse Transcription System (Promega, Mannheim, Germany). Of the resulting 20 μl of cDNA, 0.5 μl were used per semi-quantitative PCR reaction. Unless indicated otherwise, 20 μl PCR reaction mix contained 1 × Taq buffer (5PRIME; VWR International, Darmstadt, Germany), 0.2 mM dNTPs, 2 pmol sense and antisense primers, 1 M betain, and 0.2 μl 5PRIME Taq DNA polymerase (VWR International); for amplification of steroid hormone receptor cDNAs, 0.1 μl BioTherm Taq DNA polymerase (Genecraft, Lüdinghausen, Germany) was used. All programs started with a denaturation step at 95°C for 4 min and terminated with an elongation step at 72°C for 10 min. For amplification of dPRL cDNA, Hot Start Taq DNA polymerase (0.1 μl) was used with the corresponding buffer (Qiagen, Hilden, Germany) and a denaturation step at 95°C for 15 min. Specific amplification conditions and primer sequences are given in Table 1. PCR products were resolved in 2% agarose gels, stained with SYBR Gold (Molecular Probes, Invitrogen) and visualized in a Typhoon 8600 Imager (Amersham Biosciences, Freiburg, Germany).

**Table 1:** Oligonucleotide Primers and Amplification Conditions for RT-PCR

**Amplified Product**	**Forward/Reverse Primer**	**Amplification Conditions**	**Product Size**	**Genbank Accession No**.
DEPP (C10orf10)	5'-AGGTCCCGGCTTCTGCTCTCC-3' 5'-CCCATGGGCTTGCTGCTGTC-3'	20 cycles 95°C 30 sec, 60°C 30 sec, 72°C 45 sec	434 bp	NM_007021
dPRL (decidua-specific PRL transcript)	5'-GAGACACCAAGAAGAATCGGAACATACAGG-3' 5'-TCGGGGGTGGCAAGGGAAGAA-3'	25 or 30 cycles 94°C 45 sec, 57°C 45 sec, 72°C 45 sec	416 bp	M58594, GQ305133
ERα (ESR1)	5'-CCCGCCGGCATTCTACA-3' 5'-ACATTCTCCCTCCTCTTCGGTCTT-3'	30 cycles 95°C 30 sec, 52°C 30 sec, 72°C 45 sec	359 bp	NM_000125
GAPDH	5'-GGAGTCCACTGGCGTCTTCAC-3' 5'-GAGGGGCCATCCACAGTCTTCT-3'	20 cycles 95°C 30 sec, 65°C 30 sec, 72°C 30 sec	287 bp	NM_002046
HEF-1 (NEDD9)	5'-AACCGGGTGAAGCTTCTGATTGG-3' 5'-GCTGATGAGGGAGGGATGTCGTAT-3'	25 cycles 95°C 45 sec, 62°C 45 sec, 72°C 60 sec	416 bp	NM_006403
IGFBP-1	5'-TGCTGCAGAGGCAGGGAGCCC-3' 5'-AAGGATCCTCTTCCCATTCCA-3'	25 cycles 95°C 30 sec, 63°C 30 sec, 72°C 90 sec	378 bp	NM_000596
ITGA6	5'-AGCAAGGCAGATGGAATAATGTGA-3' 5'-AGGCCGGGATCTGAAAATAGTT-3'	25 cycles 95°C 45 sec, 55°C 45 sec, 72°C 60 sec	329 bp	NM_000210
KAI-1 (CD82)	5'-AGAAGTGGGCCCTGTGACC-3' 5'-TTGCCCATGTTGAAGTAGAAGAG-3'	25 cycles 95°C 30 sec, 58°C 30 sec, 72°C 90 sec	396 bp	NM_002231
p53 (TP53)	5'-TTCCACGACGGTGACACGCTT-3' 5'-GTAGCTGCCCTGGTAGGTTTTCTG-3'	25 cycles 95°C 30 sec, 57°C 30 sec, 72°C 45 sec	381 bp	NM_000546
PR-B (PGR)	5'-GCCACATTCAACACCCACTTTCTC-3' 5'-GCCTCCAGCACCCCTTGTAGC-3'	30 cycles 95°C 45 sec, 52°C 45 sec, 72°C 60 sec	411 bp	NM_000926
PR-B/-A	5'-TCCACGTGCCTATCCTGCCTCTCA-3' 5'-GCCGTCACCGCCGCTTCC-3'	30 cycles 95°C 45 sec, 61°C 45 sec, 72°C 60 sec	412 bp	NM_000926
PR-LBD	5'-GGACATGACAACACAAAACCTGAC-3' 5'-GTGCCCGGGACTGGATAAAT-3'	30 cycles 95°C 45 sec, 52°C 45 sec, 72°C 60 sec	598 bp	NM_000926
Tissue Factor (F3)	5'-TACTTGGCACGGGTCTTCTCCTAC-3' 5'-TTCTCCTGGCCCATACACTCTACC-3'	20 cycles 95°C 30 sec, 60°C 30 sec, 72°C 45 sec	431 bp	NM_001993

### Western blot

Whole cells extracts were prepared in RIPA buffer (10 mM Tris-HCl pH 7.4, 150 mM NaCl, 1 mM EDTA, 1% sodium deoxycholate, 1% Triton X-100, 0.1% SDS) with Complete protease inhibitors (Roche Applied Science, Mannheim, Germany). Proteins were electrophoresed on 10% SDS-polyacrylamide gels (NuPage Bis-Tris; Invitrogen) and transferred by tank-blotting onto polyvinylidene difluoride Immobilon membranes (Millipore, Eschborn, Germany). For electrophoresis and transfer under non-reducing conditions, RIPA lysates were added to an equal volume of loading buffer without *β*-mercaptoethanol (10 mM Tris, 10% SDS, 25% glycerol, 0.01% bromophenol blue), and antioxidant was omitted from the NuPAGE transfer buffer (Invitrogen). For reducing conditions, 25% *β*-mercaptoethanol was included in the loading buffer, and electrotransfer was carried out in the presence of 0.25% antioxidant. Immunodetection was performed with the enhanced chemiluminescence system (SuperSignal; Pierce, Bonn, Germany). KAI1 was detected under non-reducing conditions (antibody TS82b, 1:500) (Diaclone, Besançon, France). GAPDH (6C5, 1:10,000) (HyTest, Turku, Finland) and p53 (DO-1, 1:1,000) (Calbiochem, Darmstadt, Germany) were detected under reducing conditions. For analysis of FOXO1, cells were directly extracted into hot loading buffer, and equal volumes loaded per lane for reducing gel electrophoresis. The rabbit monoclonal FOXO1 antibody C29H4 (Cell Signaling Technology, Danvers, MA) was used at 1:1,000.

### IGFBP-1 ELISA

St-T1b cells were plated at a density of 4 × 10^5 ^cells per well in 6-well-plates. Two days later, media were changed, and cells received stimuli (8-Br-cAMP, P4 or MPA alone or in combination) or MM1 alone for 4 d. Media were changed again, all cells received stimuli, and 3 d later supernatants were harvested for measurement of IGFBP-1 secretion, and total RNA was extracted from the monolayers. Every treatment was done in triplicate wells. Secreted IGFBP-1 was measured by ELISA (Mediagnost, Reutlingen, Germany) (detection range: 0.1 – 8 ng/ml) and normalized to total RNA.

### Statistical analysis

GraphPad Prism software was used to perform one-way ANOVA, followed by Bonferroni's *post-hoc *test.

## Results

### Phenotypic characterization of the endometrial stromal cell line, St-T1b

**Figure 1:**
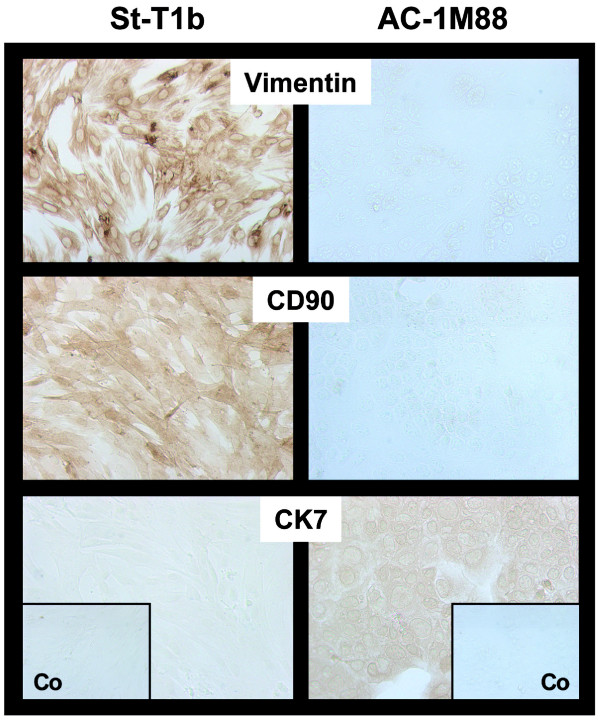
**Phenotypic markers of St-T1b cells**. St-T1b were grown to near confluency and subjected to immunocytochemical analysis using antibodies to vimentin, CD90 and cytokeratin-7 (CK7). The trophoblast-derived cell line AC-1M88 was stained alongside as a control. Control insets (Co) shows staining with omission of the primary antibody (original magnification, 100×).

St-T1b cells were initially characterized approximately 30 passages after immortalization with hTERT. First, we examined cell type-specific antigen expression of these fibroblast-derived cells. Vimentin and CD90 were used as fibroblast markers and contrasted to cytokeratin-7 (CK7) as an epithelioid marker (Fig. 1). St-T1b cells were found to display the appropriate phenotype being vimentin^+^/CD90^+^/CK7^-^. The trophoblast-derived cell line AC-1M88 served as a positive control for CK7 staining and lack of vimentin and CD90 immunoreactivity.

### Characteristics of St-T1b cells: response to cAMP

**Figure 2:**
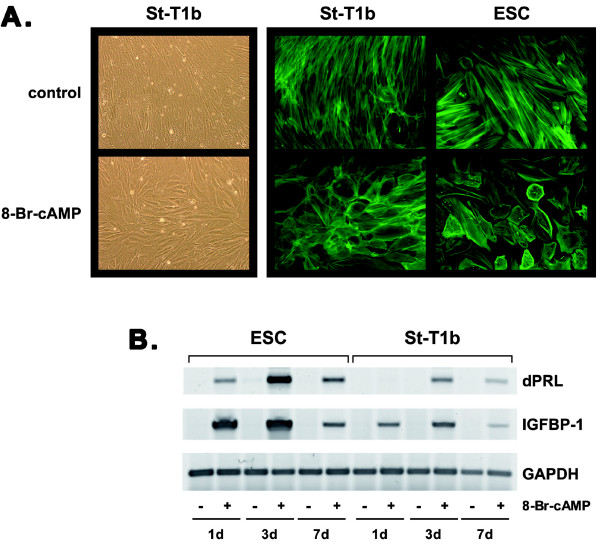
**Morphological and biochemical decidualization of St-T1b cells**. **A**) The *left panel *shows phase contrast microphotographs of untreated (control) or decidualized St-T1b cells after 7 d treatment with 8-Br-cAMP (magnification, 100×). *Right panel*: St-T1b cells and primary ESC were left untreated or exposed to 8-Br-cAMP for 4 d before staining with FITC-phalloidin to visualize the cytoskeleton (200×). **B**) St-T1b cells and ESC were cultured for 1, 3, or 7 d in the absence or presence of 8-Br-cAMP. Induction of transcripts for decidualization markers dPRL and IGFBP-1 was demonstrated by RT-PCR; GAPDH mRNA was amplified for normalization.

**Figure 3:**
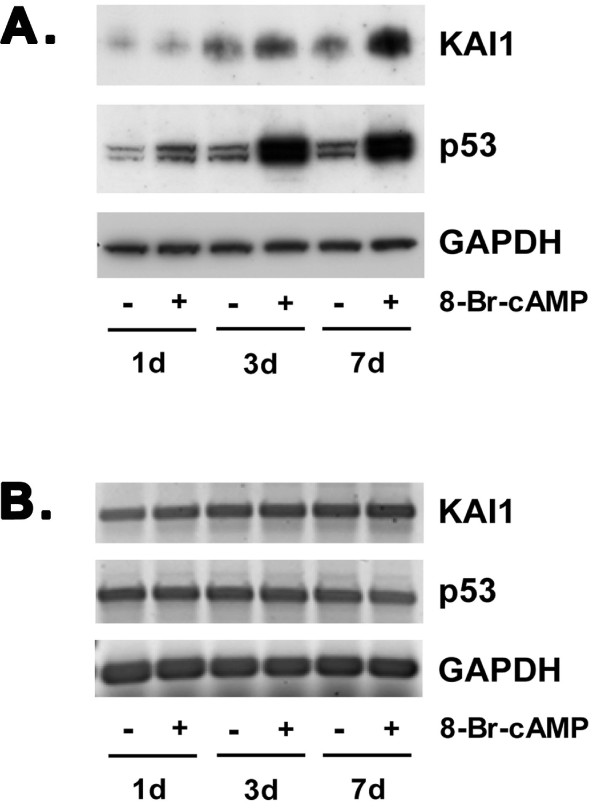
**Induction of KAI1 and p53 protein expression in decidualizing St-T1b cells**. **A**) Total protein was harvested from St-T1b cells after 1, 3, or 7 d of treatment with 8-Br-cAMP and from untreated cells. Western blot analysis was performed for KAI1 and p53, and for GAPDH as a loading control. **B**) RNA was harvested from the same cultures as described in panel A and analyzed for KAI1, p53 and GAPDH transcript levels by RT-PCR.

St-T1b cells were treated with 8-Br-cAMP, a widely established stimulus for decidualization [1]. Morphological decidualization was evident by phase contrast microscopy and upon staining of the actin cytoskeleton, showing the same reorganization from elongated to polygonal shape in St-T1b cells as seen in primary ESC (Fig. 2A). St-T1b cells were also compared to primary ESC for their ability to express typical marker genes of decidualization, namely those coding for decidual PRL (dPRL) and IGFBP-1. Maximum induction of dPRL and IGFBP-1 transcripts was seen after 3 d of cAMP treatment, and induction was maintained for at least 7 d in both cell systems (Fig. 2B). Furthermore, treatment with 8-Br-cAMP over a 7-day time-course induced a gradual increase in both p53 and KAI1 proteins but not mRNA levels (Fig. 3). In summary, the cAMP responses of St-T1b cells closely reflected those of primary ESC.

### Long-term maintenance of St-T1b cells

**Figure 4:**
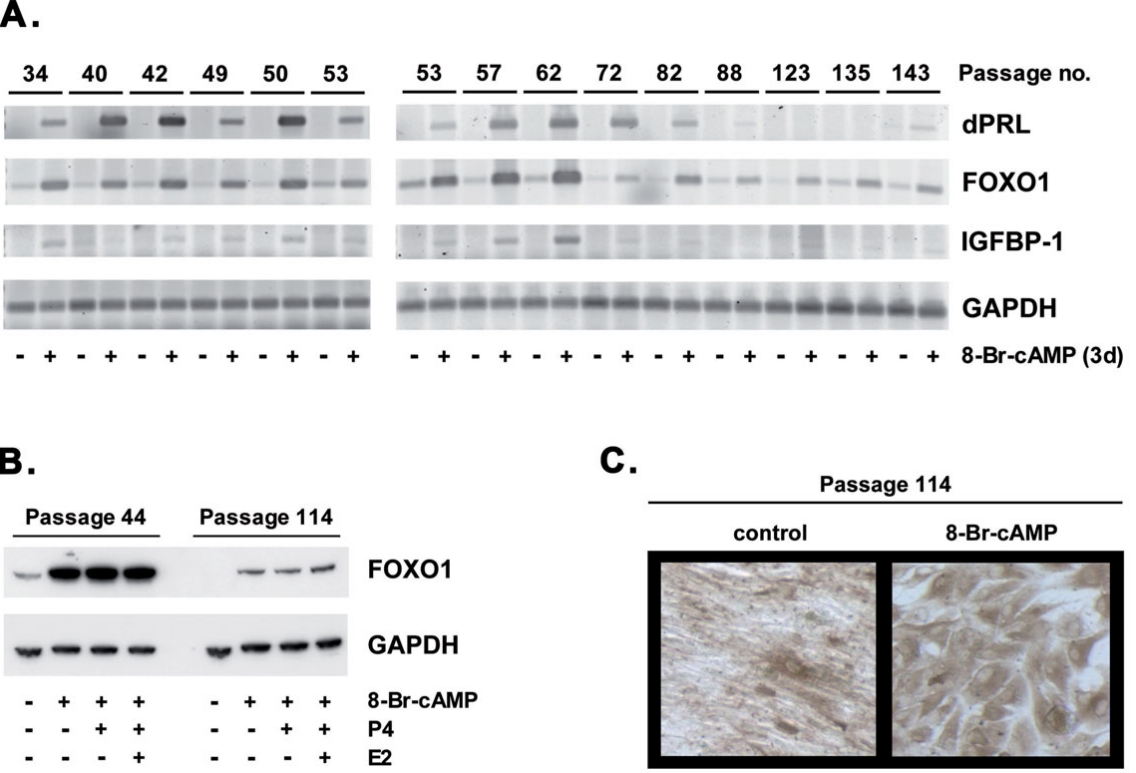
**Long-term maintenance of St-T1b cells**. **A**) St-T1b cells, obtained at passage number 30, were kept in continuous culture for the indicated number of passages and tested for their ability to decidualize in response to 3 d of 8-Br-cAMP stimulus. Transcript levels of dPRL, IGFBP-1 and FOXO1 were determined by RT-PCR; GAPDH mRNA was amplified for normalization. **B**) St-T1b cells at passage number 44 or 114 were treated with the indicated combinations of 8-Br-cAMP, P4 (1 μM) and E2 (10 nM) for 7 d before Western blot analysis for FOXO1. The blot was stripped and reprobed with GAPDH antibody. **C**) St-T1b cells at passage number 114, untreated or treated with 8-Br-cAMP for 7 d, were subjected to immunocytochemistry with vimentin antibody (original magnification, 100×).

We wondered if the cells retained their ability to decidualize in extended culture. Cells obtained at passage number 30 were therefore maintained in continuous culture for more than 113 additional passages. At intervals, a 3-day decidualization experiment was performed, measuring the relative induction of dPRL, IGFBP-1 and FOXO1 transcripts as the endpoints (Fig. 4A). The induction of dPRL mRNA was clearly detectable up to passage 82 and then began to weaken. IGFBP-1 transcripts were less robustly induced and faded around passage number 72. St-T1b displayed a cAMP-dependent induction of FOXO1 message levels throughout the entire study period with a decline at later passages. This was reflected by a decrease in cAMP-induced FOXO1 protein levels when comparing passage numbers 44 and 114. The response to cAMP was not altered by the addition of P4 or P4 plus E2 over 7 d (Fig. 4B). Morphological features of decidualization, and vimentin expression, were still maintained at passage 114 (Fig. 4C).

The doubling time of St-T1b cells was determined to be approximately 24 h. No growth crisis was apparent throughout the entire period of observation.

### Characteristics of St-T1b cells: response to progesterone

**Figure 5:**
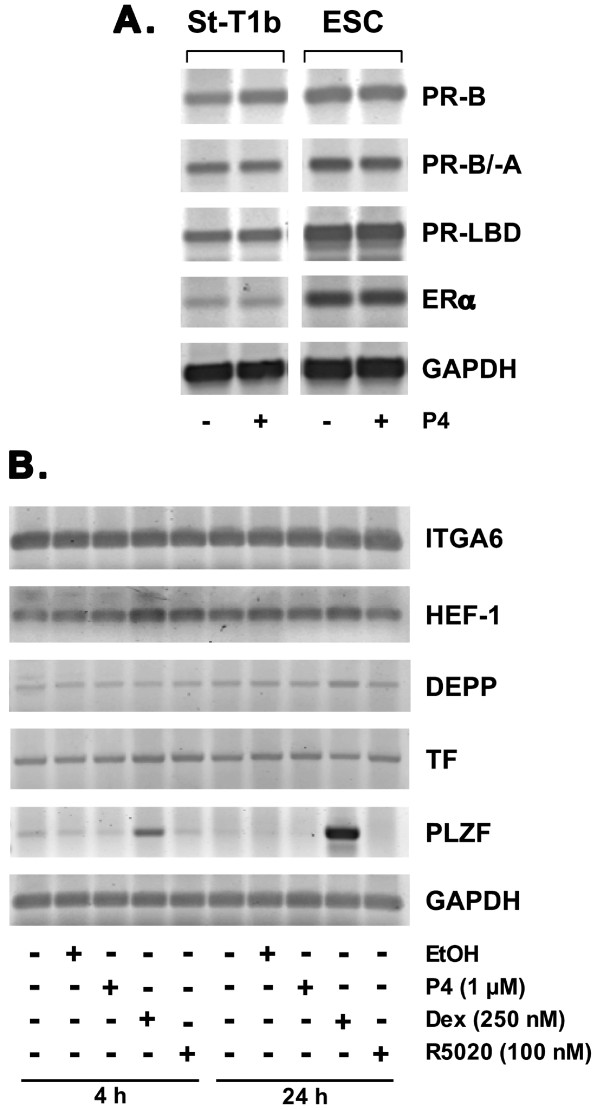
**Steroid hormone receptor expression and P4 responsiveness in St-T1b cells**. **A**) Expression of PR and ERα was analyzed by RT-PCR in St-T1b cells and ESC cultured without or with P4 (1 μM) for 4 h. PR transcripts were amplified using primers recognizing specifically PR-B, a region common to PR-B and PR-A, or the ligand-binding domain (LBD). GAPDH mRNA was amplified as a loading control. **B**) Expression of P4 target genes was analyzed in St-T1b cells by RT-PCR, after 4 or 24 h treatment with the indicated doses of P4, dexamethasone or R5020. Controls received 0.001% ethanol as a vehicle control.

We next assessed the expression of the nuclear receptors for ovarian steroid hormones, namely progesterone receptor (PR) and estrogen receptor (ER). The expression of PR was tested in the absence and presence of P4, as it has been reported that liganded PR activates its own promoter in human ESC [19]. Using primers to various regions of the PR, namely to the N-terminus specific to the full length PR-B, to a region common to PR-B and the N-terminally truncated PR-A isoform, and to the ligand-binding domain (LBD) of PR [20], expression of PR was readily detectable in both St-T1b cells and ESC. Likewise, ERα transcripts were amplified from both cell types. Transcript levels were not altered by P4 treatment (Fig. 5A). ERβ transcripts were below the limit of detection in both cell systems (data not shown).

To further explore the P4 responsiveness of St-T1b cells, we turned to a panel of established genes under the transcriptional control of the activated PR isoforms, PR-A, PR-B or both. These included tissue factor (TF; gene *F3*) and integrin α6 (*ITGA6*) as PR-B target genes [21], decidual protein induced by progesterone (DEPP; *C10orf10*) as a PR-B/PR-A target gene [22,23], HEF-1 (*NEDD9*) as a PR-A target gene [21], and promyelocytic leukemia zinc finger protein (PLZF; *ZBTB16*) as a product rapidly induced by P4 in ESC [24]. RNA was harvested after 4 and 24 h of treatment with P4, the synthetic progestin R5020 (Promegestone), or the glucocorticoid receptor (GR) agonist dexamethasone, and subjected to semiquantitative RT-PCR for the above targets (Fig. 5B). None of the transcripts was induced in response to P4 or R5020. In contrast, dexamethasone weakly up-regulated HEF-1 mRNA after 4 h but had a strong and sustained effect on PLZF mRNA expression. This is consistent with our previous observation that PLZF expression is regulated not only by P4 but also by glucocorticoids [24]. The absence of a transcriptional response to P4 prompted us to examine the expression of PR isoforms in St-T1b cells at the protein level. Neither PR-B nor PR-A were detectable by Western blotting in this cell line (data not shown). Thus, undifferentiated St-T1b cells are responsive to glucocorticoid but not to P4 as they seem to lack PR protein. In agreement, P4 treatment, in combination with E2, failed to induce morphological changes associated with decidualization in St-T1b cells (data not shown).

**Figure 6:**
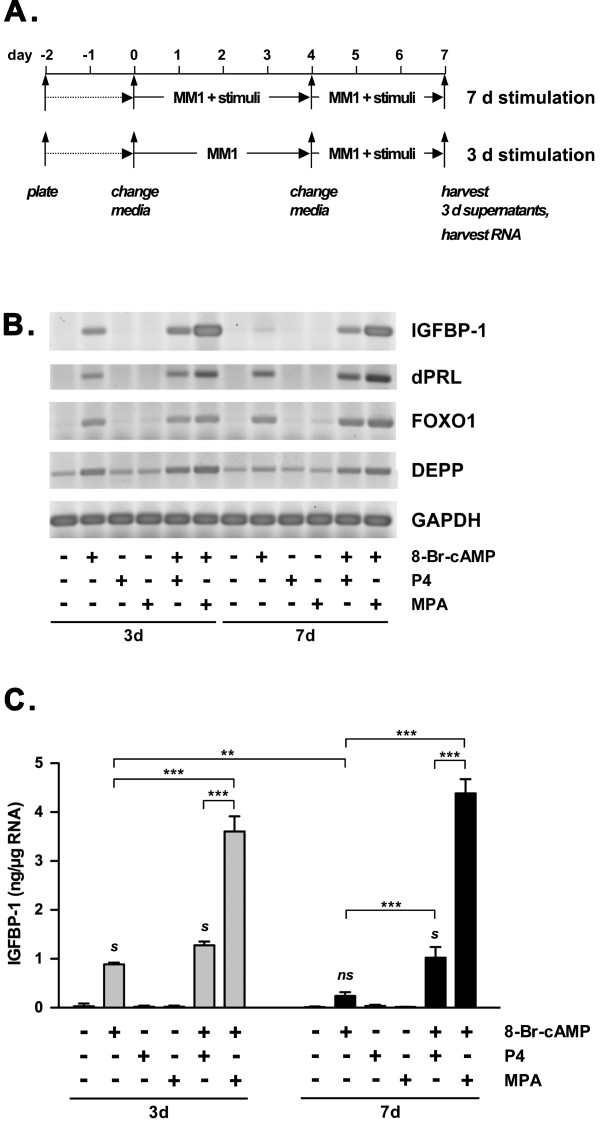
**Effect of P4 and MPA on expression of decidual products**. **A) **St-T1b cells were incubated in MM1 for 9 days as outlined in the scheme. Cultures were stimulated for 3 or 7 d with 8-Br-cAMP (0.5 mM) alone or in combination with P4 or MPA (1 μM). Supernatants were collected from the last 3 days of treatment, and RNA was harvested. **B) **RT-PCR analysis was performed for the indicated products on cells that had been stimulated for 3 or 7 d. **C) **Supernatants of the cultures described above, stimulated for 3 or 7 d, were assayed for IGFBP-1. Values represent IGFBP-1 secretion over 3 d, normalized to total RNA content of the wells. Means ± SD are shown (n = 3 wells). ANOVA followed by Bonferroni's *post-hoc *test revealed significant differences (**, *P *< 0.01; ***, *P *< 0.001; s, significantly different from controls, P4 and MPA after 3 and 7 d with *P *< 0.001; ns, not significantly different from controls, P4 or MPA after 3 and 7 d).

At physiologic concentrations, P4 has a half-life of only 2–4 h in cells, and even at 1 μM, the steroid is entirely metabolized within 18 h, whereas synthetic progestins including MPA are not metabolized [25]. Owing to its higher stability as compared to P4, MPA is frequently used in the extended treatments of ESC to induce the decidual reaction. We therefore compared the two progestins, P4 and MPA, alone and in combination with cAMP analog. St-T1b cells were treated for 3 or 7 d before supernatants and RNA were harvested (Fig. 6A). RT-PCR analysis of dPRL, IGFBP-1, FOXO1 and DEPP transcript levels revealed induction of all targets by cAMP after 3 d but not in response to progestin treatment alone for 3 or 7 days (Fig. 6B). However, different patterns of responses were observed for combined treatments. FOXO1 and dPRL mRNA were induced by cAMP after 3 and 7 d to similar degrees; this was not altered by the presence of P4 but minutely enhanced by MPA. For DEPP mRNA, on the other hand, the cAMP response seen after 3 d was reduced after 7 d, but recovered both by addition of P4 and MPA. Induction of IGFBP-1 in response to cAMP stimulation was transient, characterized by lower expression levels after 7 compared to 3 days of stimulation (see also Fig. 2). The decline in the expression of this decidual marker gene after 7 days of cAMP stimulation was entirely reversed upon co-treatment with P4 and even more so with MPA. These findings indicate a synergy between cAMP and MPA at both time points, and most importantly, the acquisition of P4-responsiveness after 7 days of exposure to cAMP. The supernatants of the above cultures were also assayed for secreted IGFBP-1. Remarkably, the expression profile seen at the mRNA level was precisely reflected at the protein level and clearly underpinned the significant reduction in cAMP-stimulated IGFPB-1 expression after 7 d, and a reversal of this reduction by added P4 at the later time point. Again, the most effective combination was that of cAMP with MPA (Fig. 6C). Secreted PRL levels were below the level of detection of the ELISA.

Taken together, cAMP-primed St-T1b cells are responsive to the synthetic progestin MPA and, upon extended induction of the decidual process, acquire responsiveness to P4.

Further experiments were undertaken to assess the role of E2. Over a 7 d stimulation, the induction of IGFPB-1, dPRL and FOXO1 expression obtained with cAMP alone, cAMP plus P4, or cAMP plus MPA was not enhanced by E2. No induction was obtained with steroids alone. Moreover, morphology of the cells was not altered by the addition of E2. Western blot analysis of the samples shown in Fig. 4B revealed no induction of PR protein in decidualized cells by E2 (data not shown).

## Discussion

Validated stable ESC lines are in demand to overcome the limitations in obtaining primary endometrial tissues for cell culture experimentation. In addition to an extended lifespan, the ability to undergo decidualization is of foremost importance in ESC lines. Here we characterize the St-T1b cell line that was generated from primary human ESC by introducing hTERT. The line has been maintained in continuous culture for more than 150 passages now without signs of crisis. It displays the typical morphology of proliferative endometrial fibroblasts and expresses the appropriate lineage-specific markers like vimentin and CD90 while being CK7-negative. In response to cAMP stimulation, the cells decidualize as judged by morphological alteration and activation of dPRL, IGFBP-1 and FOXO1 expression. Furthermore, as we have shown previously for primary ESC, these cells respond to cAMP by up-regulating KAI1 and p53 proteins [26,4,6,5]. A widely used protocol for decidualization is treatment of ESC with E2 plus P4 for up to 14 d [27-30]. Furthermore, P4 serves to enhance the action of cAMP analog as a decidualization stimulus [1]. A stable ESC line that expresses PR and displays P4-responsiveness would therefore be highly desirable. In St-T1b cells, however, despite the presence of PR transcripts, no morphological or biochemical decidualization was seen in response to even prolonged P4/E2 treatment (data not shown). Further, a range of PR target genes was not activated in St-T1b cells upon short-term P4 stimulation, and PR protein was not detectable. We have previously observed lack of a P4 response in an ESC line immmortalized with SV40 large T antigen [9]. Likewise, ESC cells immortalized with a temperature-sensitive SV40 large T antigen could readily be stimulated to decidualize in response to cAMP analog, but not P4 [31]. Loss of PR expression with time in culture is also seen in primary ESC [23]. It thus appears that PR expression does not offer a selective advantage to ESC in long-term culture.

On the other hand, an ESC line immortalized with telomerase was reported to mount a normal progestational response [10]. However, it has to be noted that neither expression of PR protein nor a response to P4 were specifically assessed in this cell line; rather it was shown that the cells decidualize in response to E2 plus MPA, a progestin capable of activating the androgen receptor (AR) and GR in addition to PR [32,25]. For another telomerase-immortalized ESC line, SHT290, a long sequential treatment protocol with epidermal growth factor (EGF), E2 and MPA was employed to induce decidualization [11]. An SV40-transformed ESC line, HIESC, was shown not only to decidualize upon combined treatment with cAMP plus MPA, but also to express PR as determined by Western blot analysis [33]. The particular PR antibody used in that study, however, yields non-specific bands and may cause misleading results [20]. These aforementioned studies suggest the possibility that AR rather than PR mediates the effect of MPA on decidualization in these cell lines. Of note, we recently demonstrated a non-redundant role of AR in decidualizing ESC. Activation of AR by dihydrotestosterone (DHT) enhanced the decidual response triggered by cAMP plus P4 signaling [34].

Here, we demonstrate for the first time a response to natural P4 in an immortalized ESC line. The transient induction of IGFBP-1 expression and secretion by cAMP, declining after 7 days of treatment, was completely recovered by addition of P4. Our findings on regulation of IGFBP-1 expression precisely reflect what has been described before in primary ESC: cAMP alone rapidly induces decidualization but is not sufficient to sustain the decidual phenotype over an extended period of time. The long-term maintenance of decidualization is achieved by the addition of P4 to cAMP-primed ESC, and is even further enhanced by DHT [34]. This combinatorial effect of cAMP, P4 and DHT might explain the strong effect seen in our system by the combination of cAMP and MPA, suggesting that MPA acts through both the PR and the AR.

While we failed to detect PR protein by Western blot analysis in decidualized St-T1b cells, in the absence or presence of E2, the receptor may still be present at a low level that is sufficient to elicit a response. Alternatively, the P4 signal may be transduced through other, still enigmatic, non-classical receptors [35].

It is remarkable that a decidual product believed to be exquisitely dependent on P4 stimulation, DEPP, was instead induced by 8-Br-cAMP in St-T1b cells. A significant overlap of products induced by cAMP analog with those induced by P4 treatment had also been revealed by gene expression profiling of primary ESC [36], underpinning the concept of intricate crosstalk between the two signaling pathways, and partial redundancy, in decidualization [1].

## Conclusion

We have characterized a telomerase-immortalized ESC line, St-T1b, that displays many features of primary ESC. Morphological and biochemical decidualization was achieved by cAMP treatment. While the cells did not respond to short-term P4 treatment by up-regulation of established P4 target genes, we could demonstrate that the cells acquired P4-responsiveness upon prolonged treatment with cAMP, and that the steroid supported maintenance of the cAMP-induced decidual phenotype. We therefore consider this cell line a valid substitute for primary ESC in the investigation into decidual transformation and function.

## List of abbreviations

AR, androgen receptor; cAMP, 3'-5'-cyclic adenosine monophosphate; CK7, cytokeratin-7; DEPP, decidual protein induced by progesterone; DHT, dihydrotestosterone; dPRL, decidual prolactin; E2, 17*β*-estradiol; ESC, endometrial stromal cells; ERα, estrogen receptor-α (ESR1); ERβ, estrogen receptor-β (ESR2); GR, glucocorticoid receptor (nuclear receptor subfamily 3, group C, member 1; NR3C1); hTERT, human telomerase reverse transcriptase; MPA, medroxyprogesterone acetate; P4, progesterone; PR, progesterone receptor (PGR); PLZF, promyelocytic leukemia zinc finger; RT-PCR, reverse transcriptase polymerase chain reaction

## Competing interests

The authors declare that they have no competing interests.

## Authors' contributions

AS and KR carried out cell culture experiments, immunocytochemistry, RT-PCR and immunoblot analyses, SW performed immunoassays, HMS participated in the design of the study, JJB prepared primary cells for immortalization and helped to draft the manuscript, AMB participated in the design of the study and drafting the manuscript, BG designed and coordinated the study and wrote the manuscript. All authors read and approved the final manuscript.
